# Focal segmental glomerulosclerosis with a mutation in the *mitochondrially encoded NADH dehydrogenase 5* gene: A case report

**DOI:** 10.1016/j.ymgmr.2023.100963

**Published:** 2023-03-09

**Authors:** Tsukasa Naganuma, Toshiyuki Imasawa, Ikuo Nukui, Masakiyo Wakasugi, Hiroshi Kitamura, Yukiko Yatsuka, Yoshihito Kishita, Yasushi Okazaki, Kei Murayama, Yoshimi Jinguji

**Affiliations:** a Division of Nephrology, Department of Internal Medicine, Yamanashi Prefectural Central Hospital, 1-1-1 Fujimi, Kofu, Yamanashi 400-0027, Japan; b Department of Nephrology, National Hospital Organization Chiba-Higashi National Hospital, 673 Nitona-cho, Chuoh-ku, Chiba-city, Chiba 206-8712, Japan; c Department of Clinical Pathology, National Hospital Organization Chiba-Higashi National Hospital, 673 Nitona-cho, Chuoh-ku, Chiba-city, Chiba 206-8712, Japan; d Diagnostics and Therapeutics of Intractable Diseases, Intractable Disease Research Center, Graduate School of Medicine, Juntendo University, 2-1-1, Hongo, Bunkyo-ku, Tokyo 113-8421, Japan; e Department of Life Science, Faculty of Science and Engineering, Kindai University, 3-4-1 Kowakae, Higashiosaka, Osaka 577-8502, Japan; f Center for Medical Genetics, Department of Metabolism, Chiba Children's Hospital, 579-1, Heta-cho, Midori-ku, Chiba 266-0007, Japan

**Keywords:** Focal segmental glomerulosclerosis, Mitochondrial nephropathy, NADH dehydrogenase 5, Podocyte, Case report, ND5, NADH dehydrogenase 5, *MT-ND5*, mitochondrially encoded ND5, OXPHOS:, oxidative phosphorylation, MELAS, mitochondrial encephalomyopathy, lactic acidosis, and stroke-like episodes, FSGS, focal segmental glomerulosclerosis, GSECs, granular swollen epithelial cells, AiDIVs, age-inappropriately disarranged and irregularly sized vascular smooth muscle cells, ReCPos, red-coloured podocytes, ATP, adenosine triphosphate, MRC, mitochondrial respiratory chain, mtDNA, mitochondrial DNA, nDNA, nuclear DNA, Cr, creatinine, sCr, serum creatinine, eGFR, estimated glomerular filtration rate, COX IV, cytochrome *c* oxidase subunit 4

## Abstract

NADH dehydrogenase 5 (ND5) is one of 44 subunits composed of Complex I in mitochondrial respiratory chain. Therefore, a *mitochondrially encoded ND5* (*MT-ND5*) gene mutation causes mitochondrial oxidative phosphorylation (OXPHOS) disorder, resulting in the development of mitochondrial diseases. Focal segmental glomerulosclerosis (FSGS) which had podocytes filled with abnormal mitochondria is induced by mitochondrial diseases. An *MT-ND5* mutation also causes FSGS. We herein report a Japanese woman who was found to have proteinuria and renal dysfunction in an annual health check-up at 29 years old. Because her proteinuria and renal dysfunction were persistent, she had a kidney biopsy at 33 years of age. The renal histology showed FSGS with podocytes filled with abnormal mitochondria. The podocytes also had foot process effacement and cytoplasmic vacuolization. In addition, the renal pathological findings showed granular swollen epithelial cells (GSECs) in tubular cells, age-inappropriately disarranged and irregularly sized vascular smooth muscle cells (AiDIVs), and red-coloured podocytes (ReCPos) by acidic dye. A genetic analysis using peripheral mononuclear blood cells and urine sediment cells detected the m.13513 G > A variant in the *MT-ND5* gene. Therefore, this patient was diagnosed with FSGS due to an *MT-ND5* gene mutation. Although this is not the first case report to show that an *MT-ND5* gene mutation causes FSGS, this is the first to demonstrate podocyte injuries accompanied with accumulation of abnormal mitochondria in the cytoplasm.

## 1 Introduction

Mitochondrial diseases/disorders are rare and occur every 1 in 5000 births [1]. Mitochondria play a key role in the biosynthesis of adenosine triphosphate (ATP), the main energy source of cells, through oxidative phosphorylation (OXPHOS) using the mitochondrial respiratory chain (MRC) complex. Given that the genes related to the MRC complex are encoded in mitochondrial DNA (mtDNA) and nuclear DNA (nDNA), mitochondrial diseases can occur in cases of mtDNA or nDNA mutations [2,3]. As ATP is mandatory for all cells that require energy, the clinical phenotypes of mitochondrial diseases are versatile and are expressed as encephalopathy [4], myopathy [5], cardiomyopathy [6], hepatopathy [7], deafness [8], and diabetes mellitus [9]. Mitochondrial nephropathy, which occurs because of a mitochondrial disorder in nephrons, has also been reported [[10], [11], [12]].

Focal segmental glomerulosclerosis (FSGS) is a histological term, rather than a specific disease category and diagnosed by the presence of sclerosis in parts of at least one glomerulus in the kidney biopsy specimen [13]. It can be classified into primary, secondary, genetic, and unknown forms of FSGS [14]. Among causative genes of mitochondrial nephropathy, m.3243A > G pathogenic variant in *MT-TL1* gene on mtDNA, which encodes mitochondrial transfer RNA leucine 1, is majority [12]. The m.3243A > G mutation leads to genetic cause of FSGS with accumulation of abnormal mitochondria in podocytes [11,15]. Because podocyte injury plays a key role in the pathogenesis of FSGS [[16], [17], [18]], impaired mitochondrial function in podocytes should induce FSGS [[19], [20], [21], [22]].

The pathogenic variants in *mitochondrially encoded NADH dehydrogenase 5* (*MT-ND5*) gene cause mitochondrial diseases such as mitochondrial encephalomyopathy with lactic acidosis and stroke-like episodes (MELAS) [[23], [24], [25]]. ND5 is one of the 44 subunits of mitochondria respiratory complex I. The pathogenic variants in *MT-ND5* gene also cause FSGS [[26], [27], [28], [29]]. However, in all cases with FSGS lesions presented in the past reports, abnormal mitochondria or their accumulation were not identified in podocytes. In another recent report of three cases with nephropathy due to *MT-ND5* gene mutation, one of the cases showed FSGS lesion. The authors of this report concluded that FSGS lesion of their case was secondary [28]. The pathogenesis of the secondary FSGS is considered to involve intraglomerular hypertension caused by hypertension or obesity, by adaptation to nephron loss due to progression of glomerulosclerosis or by a low nephron number due to low birth weight [30,31]. Therefore, it has not been evident whether genetic FSGS occurs with a pathogenic variant in *MT-ND5* gene. Here, we report the case with an *MT-ND5* mutation with apparent FSGS lesions. In this case, foot process effacement and cytoplasmic vacuolization in podocytes with accumulation of abnormal mitochondria were observed.

## 2 Case presentation

This case occurred in a Japanese woman born at 39 weeks of gestation with a birth weight of 3210 g. She had no remarkable medical history. Annual health check-ups showed no proteinuria on urinalysis until age 29. At 29 years of age, the annual health check-up revealed proteinuria (1+) for the first time using a urine dipstick and a high serum creatinine (sCr) level of 1.01 mg/dL. Her estimated glomerular filtration rate (eGFR) using the Japanese Eq. [32] was calculated to be 54.0 mL/min/1.73 m^2^. At the same check-up, she was also found to have a slight hearing disturbance. Additionally, she had a headache once a week from 30 years of age and was diagnosed by a neurologist with migraine, uncontrolled with triptan. Her health check-up at 32 years of age again showed proteinuria (1+) and increased sCr (1.06 mg/dL). Hematuria has never been noted before. At 33 years of age, she was referred to the Yamanashi Prefectural Central Hospital by her primary doctor to examine the reason for her proteinuria and decreased eGFR. At the first visit, proteinuria (1.09 g/gCr), elevated sCr (1.28 mg/dL), and elevated uric acid (7.5 mg/dL) were detected. Febuxostat and sodium bicarbonate were prescribed for the treatment of hyperuricemia. Because of the continued presentation of proteinuria and decreased renal function, she was admitted for a kidney biopsy, at 6 months following the initial visit.

Upon admission, her blood pressure was 114/70 mmHg. Physical examination showed a height of 152 cm, body weight of 43.8 kg, and body mass index of 19.0. No crackles or murmurs were detected on chest auscultation. No abnormal neurological findings were observed. Neither skin lesions nor pitting oedema were detected. The laboratory data on admission are summarised in Table 1, which shows decreased eGFR and proteinuria. Hematuria was not observed. In the data we measured, there were no data to suggest the presence of tubular dysfunction such as Fanconi's syndrome or distal tubular acidosis. The long × short axis of the kidneys measured 101 × 38 mm on the left and 104 × 50 mm on the right, indicating no renal atrophy. The electrocardiogram test result was normal, and echocardiography revealed normal cardiac function. The standard pure-tone hearing test showed a right-ear value of 38.8 dB and a left-ear value of 40.0 dB. She was therefore diagnosed with sensorineural hearing loss by an otolaryngologist. No visual field defects were noted. Furthermore, because of hearing loss and headache symptoms, a brain magnetic resolution imaging (MRI) was also performed to examine for brain lesions, but no abnormal findings were found.

**Table 1:** Laboratory data on admission.

Blood cell count			Blood chemistry			Immunology			Urinalysis		
WBC	5400	/μL	TP	6.8	g/dL	CH50	54	U/mL	Gravity	1.01	
RBC	373	×10^4^/μL	Alb	3.9	g/dL	C3	76.1	mg/dL	pH	7.0	
Hb	11.5	g/dL	AST	21	IU/L	C4	19.3	mg/dL	RBC	1–4	/HPF
Ht	35.1	%	ALT	11	IU/L	IgG	1340.3	mg/dL	WBC	<1	/HPF
MCV	94.1	fl	LDH	186	IU/L	IgA	225.7	mg/dL	Protein	2.04	g/gCr
MCHC	32.8	%	ALP	149	IU/L	IgM	180.1	mg/dL	Glucose	(−)	
Plt	25.7	×10^4^/μL	Tbil	0.56	mg/dL	ANA	(−)		NAG	8.6	U/L
			BUN	21.8	mg/dL	M protein	(−)				
Coagulation			Cr	1.26	mg/dL	HBs Ag	(−)				
APTT	34.0	*sec*	eGFR	40.8	mL/min/1.73 m^2^	HCV Ab	(−)				
PT	99.0	%	K	5.0	mEq/L						
INR	0.97	INR	P	3.1	mg/dL						
Fib	373.0	mg/dL	UA	5.4	mg/dL						
			Tcho	255	mg/dL						
			CRP	0.01	mg/dL						
			FBS	85	mg/dL						
			HbA1c	5.3	%						

Aberrant values are underlined.

WBC, white blood cells; RBC, red blood cells; Hb, haemoglobin; Hct, haematocrit; MCV, mean corpuscular volume; MCHC, mean corpuscular haemoglobin concentration; Plt, platelet; APTT, activated partial thromboplastin time; PT, prothrombin time; INR, international normalized ratio; Fib, fibrinogen; TP, total protein; Alb, albumin; AST, aspartate aminotransferase; ALT, alanine aminotransferase; LDH, lactate dehydrogenase; ALP, alkaline phosphatase; Tbil, total bilirubin; BUN, blood urea nitrogen; Cr, creatinine; eGFR, estimated glomerular filtration rate (by the Japanese equation); K, potassium; P, phosphate; UA, uric acid; Tcho, total cholesterol; CRP, C-reactive protein; FBS, fasting blood sugar; HbA1c, haemoglobin A1c (NGSP); CH50, 50% haemolytic complement activity; C3, complement 3; IgG, immunoglobulin G; ANA, antinuclear antibody; M protein, monoclonal protein; HBs Ag, hepatitis B surface antigen; HCV Ab, hepatitis C virus antibody; NAG, *N*-acetyl glucosaminidase; HPF, high power field.

Her parents had died because of cancer (mother, breast cancer, father, colon cancer) without kidney disease or any hearing difficulty. Neither of her two older brothers had any abnormalities in kidney function, urine, or hearing. She had a 7-year-old daughter and a 5-year-old son. Her daughter's birth weight was 2840 g at 41 weeks and 2 days of gestation, and her son's birth weight was 2632 g at 39 weeks of gestation. Her daughter was born using suction delivery, whereas her son was born through normal delivery. None of her children had any urinary abnormalities or hearing difficulties, as examined during medical check-ups. However, the children sometimes complained of headaches that had not yet been diagnosed by any doctor.

**Fig. 1:**
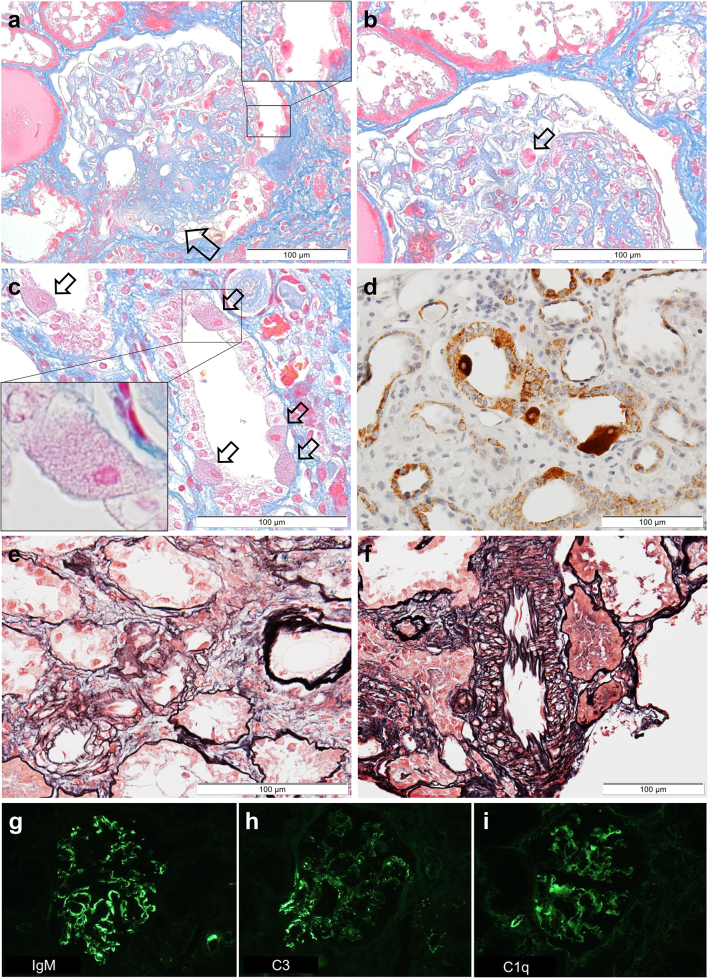
Light microscopy and immunofluorescence findings (a): The segmental sclerosis lesion at the perihilar area is indicated by an arrow. A red-coloured podocyte (ReCPo) is surrounded by a square, magnified at the top right (AZAN stain). (b): The ReCPo is indicated by an arrow (AZAN stain). (c): GSECs in the distal tubules are indicated by arrows (AZAN stain). One GSEC is magnified at the lower left. (d): Staining of COX IV emphasizes tubular cells, including many mitochondria, which correspond to GSECs. (e): In the afferent arteriole that connects to the glomerulus of (a), the sizes of the vascular smooth muscle cells are irregular, and their arrangement is disorganized, similar to those seen in older patients (PAM-HE stain). (f): Age-inappropriately disarranged and irregularly sized vascular smooth muscle cells (AiDIVs) are observed in an interlobular artery (PAM-HE stain). (g): IgM deposits are detected in glomeruli. (h): IgG deposits are detected in glomeruli. (i): C1q deposits are detected in glomeruli.

**Fig. 2:**
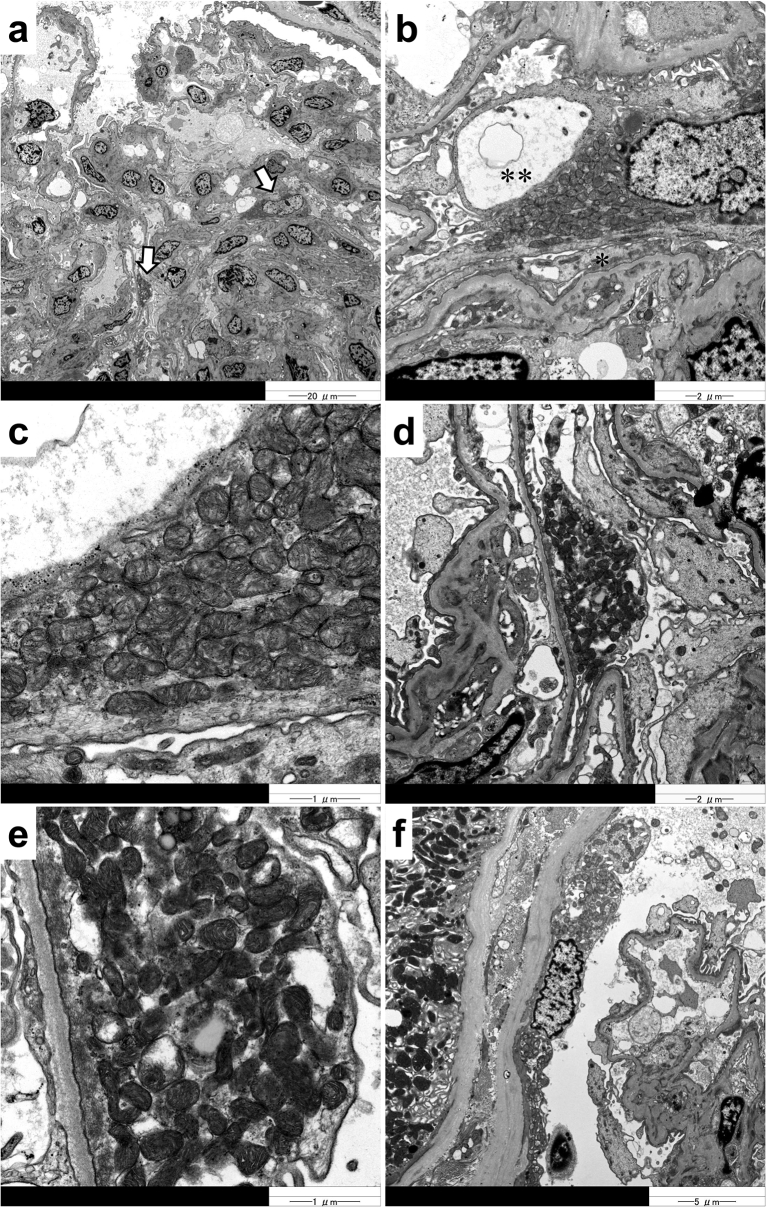
Electron microscopy findings (a): Arrows indicate podocytes filled with increased mitochondria. (b): In a podocyte, abnormally high numbers of mitochondria are seen in the cytoplasm. Foot process effacement (*) and cytoplasmic vacuolization (**) are observed in this podocyte. (c): Magnification of (b) reveals that the increased mitochondria have lost their organized cristae structure. (d): A podocyte is filled with mitochondria. The foot process of this podocyte is effaced. (e): Magnification of (d) reveals that mitochondria with disorganized cristae increase. (f): The parietal epithelial cells in Bowman's capsule are also filled with abnormal mitochondria.

A percutaneous kidney biopsy was performed under ultrasound guidance. Light microscopy photographs of renal biopsy specimens are shown in Fig. 1. Nine glomeruli were observed in the specimens. One glomerulus was globally sclerosed. Furthermore, segmental sclerosis was observed at the perihilar area in two glomeruli, compatible with the perihilar variant in the Columbia classification of FSGS (Fig. 1a) [13,30]. Interestingly, red-coloured podocytes (ReCPos), whose cytoplasm was dyed red, were observed using AZAN trichrome staining (Fig. 1a and b). Neither mesangial proliferation nor hypercellularity were detected. Interstitial fibrosis and tubular atrophy were observed in almost 30% of the interstitium. There were no abnormal findings such as thickening or lamellation of the tubular basement membrane. Characteristically, granular swollen epithelial cells (GSECs), reported to be specific to mitochondrial diseases [33,34], were markedly observed in the distal tubular cells using AZAN trichrome staining (Fig. 1c). Such GSECs were emphasised by staining with anti-cytochrome *c* oxidase subunit 4 (COX IV) antibody [mouse anti-COX IV antibody (20E8C12)] (ab14744; Abcam PLC., Cambridge, UK; Fig. 1d). COX IV staining is recognised as a loading control for mitochondria [34,35]. Furthermore, the sizes of the vascular smooth muscle cells in arterioles were irregular, and their arrangement was disorganized in a manner similar to that seen in older patients, despite the younger age (33 years) of the patient, without any medical history that may have caused arteriosclerosis (Fig. 1e). Such age-inappropriately disarranged and irregularly sized vascular smooth muscle cells (AiDIVs) were also noticeable in the interlobular arteries (Fig. 1f). Immunofluorescence analysis revealed IgM, C3 and C1q deposits in the glomeruli (Fig. 1g-i). IgG and IgA were negative in observed glomeruli. Electron microscopy analyses (Fig. 2a–e) also showed podocytes with increased mitochondria that had lost their normal cristae structure. Foot processes of such podocytes with accumulation of abnormal mitochondria were effaced (Fig. 2b and d). A vacuolization was also observed in the cytoplasm in the podocyte with mitochondrial accumulation (Fig. 2b). We also detected cells with increased abnormal mitochondria in the parietal epithelial cells of the Bowman's capsule (Fig. 2f). Although the glomerular basement membrane was partially thin at the part along with the podocytes with abnormal mitochondria, the membranes at other parts showed normal thickness (> 250 nm) without lamination or reticulation. Electron dense deposits were not observed in mesangium or glomerular basement membranes. These renal pathology findings strongly suggest genetic FSGS due to mitochondrial disease.

The serum lactate and pyruvate levels were slightly elevated at 21.0 mg/dL (normal: 3.0–17.0 mg/dL) and 1.34 mg/dL (normal: 0.30–0.94 mg/dL), respectively. The pathological finding of accumulation of abnormal mitochondria in glomerular epithelial cells and tubules, the presence of sensorineural hearing difficulty, and elevated blood lactate led us to perform a comprehensive genetic analysis of mitochondrial diseases.

**Fig. 3:**
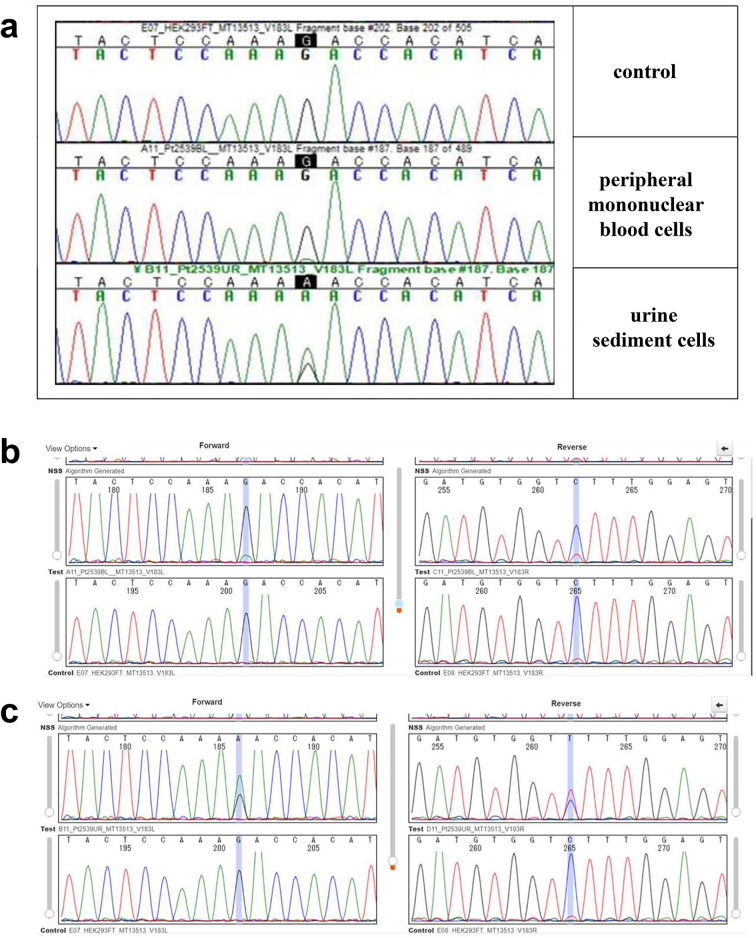
Genetic analysis (a): The electropherogram from Sanger sequencing indicates that the patient has the m.13513 G > A variant in her peripheral mononuclear blood and urine sediment cells. (b): The rate of heteroplasmy calculated by the MVF software program is 10.3% (forward: 11.6%/reverse: 9.0%) in peripheral mononuclear blood cells (upper panel: patient, lower panel: control). (c): The rate of heteroplasmy is 62.2% (forward: 63.7%/reverse: 60.7%) in urine sediment cells (upper panel: patient, lower panel: control).

After obtaining informed consent from the patient according to protocol and permission from our ethics committees, genomic DNA extracted from the patient's peripheral mononuclear blood cells and urine sediment cells was analysed by targeted resequencing coupled with next-generation sequencing, followed by Sanger sequencing. The DNA was subjected to fragmentation and library preparation using the Lotus DNA Library Prep Kit (#10001074; Integrated Device Technology, Inc., San Jose, CA, USA) according to the manufacturer's instructions. Target enrichment was performed with xGen Human mtDNA Research Panel (#1075705; Integrated Device Technology) targeting the whole mtDNA and with custom xGen® Predesigned Gene Capture Pools (Integrated Device Technology)/xGen Lockdown Probe pool (Integrated Device Technology) targeting the exons of 367 nuclear-encoded genes that cause mitochondrial diseases. The library was then sequenced on the Illumina MiSeq platform using the MiSeq Reagent Kit v2 (MS-102-2002; Illumina, Inc., San Diego, CA, USA). A pathogenic mtDNA variant of m.13513G > A was detected in both the patient's blood cells and urine sediment cells (Fig. 3). The Minor Variant Finder (MVF) software program (Thermo Fisher Scientific, Waltham, MA, USA) revealed that the heteroplasmy rates of the blood cells and urine sediment cells were 10.3% (forward: 11.6%/reverse: 9.0%) and 62.2% (forward: 63.7%/reverse: 60.7%), respectively. Therefore, we diagnosed her with renal dysfunction and proteinuria due to FSGS caused by an *MT-ND5* mutation.

## 3 Discussion and conclusions

*MT-ND5* gene encodes NADH dehydrogenase 5 (ND5), which is a subunit of mitochondria respiratory complex I. Therefore, *MT-ND5* mutations cause mitochondrial diseases such as MELAS [23,36,37], Leigh syndrome [[36], [37], [38], [39]], and Leber hereditary optic neuropathy [37]. Our case with the m.13513G > A variant in the *MT-ND5* gene showed FSGS lesions with injured podocytes that had accumulation of abnormal mitochondria in the cytoplasm. The m.13513G > A has already been confirmed as a pathogenic variant of the mitochondrial disease using MITOMAP, a human mitochondrial genome database (https://www.mitomap.org/MITOMAP).

Mitochondrial diseases cause genetic FSGS [11,12,15]. Its etiology is postulated to be caused by genetically disrupted mitochondrial function in podocytes [19,22]. As an indication of this, cases with FSGS due to m.3243A > G mutation had podocytes filled with mitochondria with abnormal cristae structure [10,11]. In the past, several cases of mitochondrial diseases due to *MT-ND5* mutations have been also reported to show FSGS lesions [[26], [27], [28], [29]]. However, in these cases, pathological findings of podocyte injuries with accumulation of abnormal mitochondria were not confirmed, leaving the possibility of secondary FSGS caused by intraglomerular hypertension [14,28,30,31]. In contrast, our present case showed apparent FSGS lesions, and we detected increased abnormal mitochondria in the glomerular podocytes that also had pathological findings indicating their injuries such as foot process effacement and the cytoplasmic vacuolization. Our patient did not have hypertension, obesity, or a history of low birth weight. Furthermore, the diameters of all glomeruli observed under light microscopy in this patient were < 250 μm (mean ± SD; 149 ± −72 μm). Therefore, we deemed the etiology of FSGS, in this case, to be a disorder of the mitochondrial OXPHOS system due to an *MT-ND5* mutation and not due to ‘secondary’ by intraglomerular hypertension [14,30,31]. The pathological picture of tubulointerstitial nephropathy with GSECs was also observed in this case. This tubulointerstitial damage may be due to the *MT-ND5* mutation, as has been reported in the past [28].

In general, mitochondrial diseases induced by mtDNA mutations vary markedly based on differences in the rates of heteroplasmy by cell type or organ [40,41]. Therefore, while the reason for the differences between the present and the previous cases with the pathogenic variant in *MT-ND5* gene could not be completely ascertained, the heteroplasmy rate of mtDNA with the pathogenic variant in podocytes might have been higher in our case than that in the previous cases.

In this case, IgM, C3, and C1q were positive in glomeruli (Fig. 1g-i). Since neither hematuria, mesangial cell proliferation, nor electron dense deposits were observed, we considered that it was unlikely that IgM nephropathy or C1q nephropathy was complicated [42,43]. On the other hand, glomerular IgM and C3 deposits frequently accompany FSGS [44]. In addition, IgM and C3 were positive in glomeruli of two out of three cases report by Bakis et al., too [28]. Therefore, IgM and C3 deposition may be related to a part of the pathogenesis of FSGS caused by *MT-ND5* mutation. Although the reason for C1q deposition in glomeruli is also not clear. Because C1q is reported to bind to mitochondria, it may be associated with the pathogenesis of FSGS by driving oxidative stress [[45], [46], [47]].

This case should be suggestive because it had several characteristic light-microscopical findings of mitochondrial nephropathy. In our case, GSECs with red-coloured cytoplasm were observed with AZAN trichrome staining. As mitochondria are dyed red by acidic dyes such as AZAN, red-coloured GSECs indicate that they abnormally include many mitochondria [33]. In addition, as COX IV is a subunit of complex IV, COX IV staining can be used to stain mitochondria [35] and may help confirm the presence of abnormally increased numbers of mitochondria [34,48]. Furthermore, age-inappropriately disarranged and irregularly sized vascular smooth muscle cells (AiDIVs) were observed in the arterioles and intralobular arteries in this case. Such findings have also been reported in other cases of mitochondrial nephropathy [12,34]. Interestingly, red-coloured podocytes (ReCPos) were detected using AZAN trichrome staining, as we recently reported in a case of mitochondrial nephropathy [49]. Like GSECs, ReCPos abnormally include many mitochondria in their cytoplasm [49]. In summary, in cases of FSGS or glomerulosclerosis with unknown aetiologies, the existence of GSECs, AiDIVs, and ReCPos should be carefully evaluated. COX IV staining may aid in the detection of abnormally increased mitochondrial counts.

We describe the weaknesses of our report. In this case, because mitochondrial disease was suspected based on the pathological findings of abnormal mitochondria, the presence of hearing loss, and high blood lactate levels, we comprehensively examined the genes associated with mitochondrial diseases. Therefore, genetic FSGS due to other gene mutation [50] cannot be completely ruled out. In addition, because genetic testing was performed only on the patient but not on other family members, it could not be investigated whether they carried the heteroplasmic mitochondrial variant. The parents were already deceased and the two healthy brothers did not consent to genetic analysis. The two her children were still under 18 years old and the patient did not wish genetic test. Furthermore, measurement of MRC enzyme activity using kidney biopsy specimens is an effective for confirming mitochondrial dysfunction [51,52]. However, it could not be performed due to the paucity of kidney specimens for this analysis.

In conclusion, this is the first case report which indicates that the pathogenic variant an *MT-ND5* gene causes accumulation of abnormal mitochondria in podocytes, which leads to forming FSGS.

## Ethics approval and consent to participate

The original study to perform a genetic analysis for mitochondrial diseases was approved by the Ethics Committee of Chiba Children's Hospital (2014-11-05). In addition, the analysis of this case was also approved by the Ethics Committee of Yamanashi Prefectural Central Hospital (2020–16). The patient provided informed consent and permission to participate in the study.

## Consent for publication

Written informed consent was obtained from the patient for the publication of this case report and accompanying images. A copy of the written consent is available for review by the editor of this journal. All authors consented to the publication of the manuscript in *BMC Nephrology*.

## Funding

This work was supported in part by the Practical Research Project for Rare/Intractable Diseases from the 10.13039/100009619Japan Agency for Medical Research and Development, AMED (19ek0109273, 20ek0109468) (http://www.amed.go.jp/en/) to TI, YO, and KM.

## Authors' contributions

TN drafted the manuscript. TI was involved in the coordination of the study and draft preparation. IN and MW provided clinical information. KH performed the pathological analysis. YY and YK performed molecular genetic studies. YO performed sequence alignment and data curation. KM was involved in the coordination of the study and data curation. YJ was involved in the coordination of the study and clinical information. All authors read and approved the final manuscript.

## Declaration of Competing Interest

The authors declare that they have no competing interests.

## Data Availability

Most of the clinical data used and analysed in this case report are presented in this manuscript. More detailed information is available from the corresponding author upon request.
